# Ocular mpox in Southern Nigeria during the 2022 and 2024 outbreaks: a report of two cases

**DOI:** 10.11604/pamj.2026.53.162.48485

**Published:** 2026-04-16

**Authors:** Chizaram Anselm Onyeaghala, Nneka Marian Chika-Igwenyi

**Affiliations:** 1 Department of Internal Medicine, University of Port Harcourt Teaching Hospital, Port Harcourt, Nigeria,; 2 Department of Internal Medicine, Alex Ekwueme Federal University Teaching Hospital, Abakaliki, Nigeria

**Keywords:** Mpox, ocular mpox, ophthalmic manifestations, surveillance, Nigeria, case report

## Abstract

There are significant knowledge gaps regarding the burden and clinical progression of ocular manifestations of mpox in Nigeria and worldwide. Here, we report two laboratory-confirmed mpox cases among young male adults with distinct ocular mpox presentations at two tertiary hospitals in southern Nigeria during the 2022 and 2024 global outbreaks. The first patient, a 28-year-old man, presented at the Alex Ekwueme Federal University Teaching Hospital in Abakaliki with a vesiculopustular rash on his nasal bridge and right lower eyelid, along with redness, pain, and itching. He subsequently developed corneal ulcers and vision problems after discharge, necessitating an ophthalmology review. The second case involved a 44-year-old man with advanced HIV, who presented with widespread vesiculo-pustular eruptions, including characteristic periorbital and orbital rashes. He presented with widespread vesiculo-pustular skin eruptions, including characteristic rashes around the periorbital and orbital areas. His condition deteriorated rapidly, leading to his death after seven days of hospitalisation. Consistent with guidance from the Nigeria Center for Disease Control and Prevention, lesional skin swabs from both patients were sent to the National Reference Laboratory in Abuja for quantitative polymerase chain reaction (qPCR), which tested positive for the monkeypox virus. Supportive care included antibiotics, antivirals, intravenous fluids, nutritional support, and skin care, as no specific mpox treatments were available at that time. Ocular disease has emerged as an increasingly common yet underreported manifestation of mpox in Nigeria since the 2022 global outbreak. Ophthalmologists should be part of the multidisciplinary team of specialists caring for mpox patients with ocular manifestations. Ongoing surveillance and research are vital to better understand eye-related complications and to improve the prevention and treatment of ocular symptoms, thereby preventing significant vision loss.

## Introduction

Mpox (formerly known as monkeypox) is an emerging viral zoonotic disease caused by the monkeypox virus (MPXV). This double-stranded DNA virus belongs to the genus *Orthopoxvirus* and the family *Poxviridae*. MPXV has two genetically distinct clades: Clade I and Clade II, with Clade II historically associated with a lower fatality rate than Clade I infections. MPXV has two genetically distinct clades: Clade I and Clade II, with Clade II historically associated with a lower fatality rate than infections of Clade I. Before the 2022 global outbreak, mpox was mainly limited to Central and Western Africa, where it had been overlooked for decades. Clade I was found in Central Africa, while Clade II was common in Western Africa [1]. Mpox typically begins with an initial prodromal phase, characterised by fever, lymphadenopathy, myalgia, fatigue, and headache. This is followed by vesiculopustular eruptions that begin on the face and spread outward in a centrifugal pattern, typically occurring 2 to 3 days after symptom onset [2]. The incubation period of MPXV generally ranges from 5 to 21 days, depending on the infecting Clade, the host's immune status, and the route of transmission [3]. Most patients with mpox recover within 2 to 4 weeks after skin eruptions appear, either spontaneously or with supportive care. However, severe illness with complications such as pneumonia, encephalitis, sepsis, secondary bacterial infections, and death can occur in the elderly, children, pregnant women, and individuals with chickenpox coinfection [4], as well as in those with weakened immune systems [5], if not properly managed.

There are notable gaps in understanding regarding the clinical features, long-term effects, and treatment options for ocular mpox. Most information on its signs and symptoms comes from case reports, and further research is needed. The 2022 global outbreak may suggest changes in the frequency and patterns of ocular mpox, which has increasingly been identified as a complication. However, it remains poorly understood in endemic mpox regions in Africa. The reported rate of eye-related symptoms in Clade II mpox infections during the 2022 outbreak ranges from 0.3% to 11%, significantly lower than the 4.1% to 23.1% observed in Clade I mpox infections before 2022. During the 2017 mpox resurgence in Nigeria, caused by Clade IIb, the most common ocular feature was the characteristic rash in the periorbital and orbital areas. However, Clade I reports often list conjunctivitis as the most frequent eye issue, usually associated with more severe illness [6]. Serious eye complications such as corneal ulcers, perforations, or episcleritis have also been reported, sometimes resulting in substantial vision loss [7]. Given the ongoing increase in mpox cases in Africa, this study aimed to report two laboratory-confirmed cases of mpox with distinct ocular manifestations, managed during the 2022 and 2024 outbreaks in southern Nigeria. This paper aims to offer valuable lessons for the inclusion of ophthalmologists on the mpox clinical team, particularly in cases of ocular mpox.

## Patient and observation

### Case 1

**Patient information:** a 28-year-old male artisan presented to Alex Ekwueme Federal University Teaching Hospital in Abakaliki, Nigeria, with a seven-day history of high-grade fever, a generalised throbbing headache, sore throat, and body pain. Three days later, he developed a solitary vesicular rash on a hypertrophied scar on his left shoulder, which then spread to his scalp, face, trunk, and groin. Initially, the rash was itchy, but later became painful genital ulcers. About two days later, he noticed a similar rash on his lower eyelid, which became very painful and was accompanied by redness and decreased vision. He was recently released from a correctional facility where he had close contact with inmates with similar lesions.

**Figure 1:**
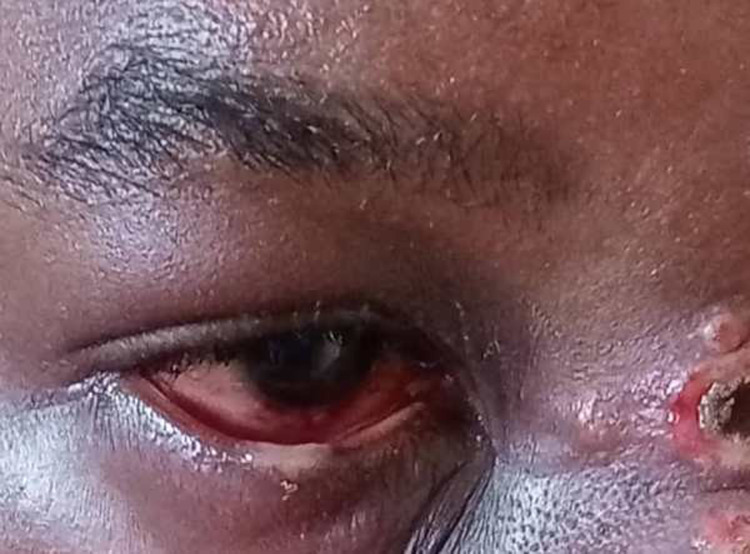
ocular mpox patient showing unilateral conjunctivitis with blepharitis

**Clinical findings:** during the general examination, he was febrile (38.2°C), not pale, anicteric, and dehydrated, with right-sided conjunctival injection and enlarged cervical and inguinal lymph nodes. His pulse rate was 92 beats per minute, blood pressure was 110/70 mmHg, respiratory rate was 21 breaths per minute, and oxygen saturation was 98% on room air. Skin examination revealed generalised vesiculo-pustular skin lesions. Gross eye examination showed bilateral red conjunctivae (Figure 1). Other systemic examination was unremarkable.

**Diagnostic assessment:** a skin swab and a crust specimen were submitted for quantitative PCR at the National Reference Laboratory of the Nigeria Centre for Disease Control and Prevention, and both tested positive for the monkeypox virus. The HIV screening test was negative.

**Therapeutic interventions:** he was admitted to the isolation ward and received supportive care, including fluid management with normal saline, empirical broad-spectrum antibiotics (IV ceftriaxone and azithromycin), intravenous valacyclovir, and antipyretics.

**Follow-up and outcomes of interventions:** the patient was discharged after a 10-day hospital stay once the rash resolved. During follow-up, he developed a corneal ulcer with worsening vision, which required a referral to the ophthalmology department for further specialised care.

**Patient perspective:** he was grateful for the care and treatment he received for his illness.

**Informed consent:** consent was obtained for this publication.

### Case 2

**Patient information:** a 44-year-old male security officer presented to the University of Port Harcourt Teaching Hospital in Port Harcourt with a 9-day history of fever and widespread skin rashes. The skin lesions were first noted on the genitals and rapidly spread to the limbs, trunk, scalp, and face, including the upper and lower eyelids of both eyes. Over a short period, the lesions became painful and necrotising, and the patient reported decreased vision in both eyes. There was a history of unprotected sexual intercourse with an acquaintance about 10 days before the first lesions appeared.

**Clinical findings:** during physical examination, he appeared acutely ill, febrile, dehydrated, with enlarged axillary lymph nodes and generalised body swelling. Vital signs indicated an axillary temperature of 38.9°C, a pulse rate of 110 beats per minute, a blood pressure of 90/60 mmHg, a respiratory rate of 36 breaths per minute, and an oxygen saturation of 86% on room air. Skin examination showed widespread necrotising skin lesions involving both eyes. He was conscious but lethargic, with crepitations heard in both lung fields on auscultation and vague abdominal tenderness.

**Diagnostic assessment:** skin samples were sent to the National Reference Laboratory of the Nigerian Centre for Disease Control and Prevention and tested positive for both monkeypox virus and varicella-zoster virus. HIV-1 and HIV-2 screening was positive, with a CD4 count of less than 200 cells/mm^3^.

**Therapeutic interventions:** he was admitted to the infectious diseases treatment center and commenced on broad-spectrum intravenous antibiotics (ceftriaxone and metronidazole), received fluid management with normal saline, oxygen therapy, intravenous acyclovir, antiretroviral therapy (tenofovir disoproxil fumarate, lamivudine, and dolutegravir), antipyretics (paracetamol), nutritional support, and skin care.

**Follow-up and outcomes of interventions:** the patient gradually deteriorated and ultimately died after 7 days in the hospital.

**Informed consent:** the wife provided consent for this publication.

## Discussion

Ocular disease has emerged as an increasingly common yet underreported complication of mpox infection. This paper describes two young adult males who developed ocular mpox while receiving care at two tertiary hospitals in southern Nigeria during the 2022 and 2024 outbreaks. The development of a corneal ulcer and decreased vision in the immediate post-discharge period of the first case underscores the importance of early ophthalmic intervention in mpox case management to prevent sight-threatening complications.

The spectrum of ocular signs associated with mpox ranges from mild symptoms, including blepharitis, conjunctivitis, focal conjunctival lesions, photophobia, and early-diagnosed keratitis, to more serious complications, such as corneal scarring and vision loss in later stages [7]. This aligns with the diverse eye-related signs observed in our two patients: the first had conjunctivitis, while the second developed distinctive periorbital and orbital rashes. In a retrospective study by Ogoina *et al*. 25% of hospitalized mpox patients presented with periorbital and orbital rashes, suggesting that vesiculo-pustular rashes may be the most common presentation of ocular mpox in Nigeria [6]. However, conjunctivitis is the most reported eye problem in studies from regions affected by Clade I mpox, and it has been linked to more severe disease progression.

Although mild and early complications are usually associated with visual and symptom recovery, our first case, who had conjunctivitis, developed a corneal ulcer and impaired vision due to a lack of early ophthalmic review during the hospital stay. This highlights the increasing importance of prompt referral to or invitation of ophthalmologists for patients with ocular mpox to prevent vision loss [8].

The exact cause of ocular symptoms in patients with mpox remains unclear, whether it results from systemic spread of the virus during the early viraemic phase or from self-inoculation into the eyes. Additionally, emerging data suggest that MPXV genomes can persist in both external and internal ocular fluids, though the significance of this finding remains uncertain. While it is proposed that MPXV may enter conjunctival secretions through the plasma compartment, the fact that skin lesions often appear before ocular symptoms indicates that self-inoculation may be the primary cause. Therefore, healthcare professionals should implement preventive measures to protect patients' eyes during acute mpox infection, such as advising patients on proper infection-prevention strategies, including hand hygiene and avoiding eye contact.

Ocular mpox should be considered in any patient with recent or past skin lesions indicative of mpox or laboratory-confirmed mpox presenting with new eye symptoms. In addition to diagnosing and treating ocular mpox, patients should also be assessed for secondary bacterial (Staphylococcus aureus, Pseudomonas aeruginosa, syphilis), viral (herpes simplex virus, varicella-zoster virus), or fungal (Aspergillus spp.) co-infections, which may complicate or mimic ocular mpox [9].

Managing ocular mpox requires collaboration between infectious disease specialists and ophthalmologists.

In particular, ophthalmologists will perform a thorough evaluation of the affected ocular structures (e.g., the cornea and conjunctiva) and monitor the patient's condition and disease extent, especially in cases with vision changes, eye pain, or worsening redness. Although there is no definitive or established treatment for ocular mpox, oral tecovirimat has been used despite a lack of pharmacokinetic data on its penetration into the ocular surface or deeper ocular structures. Additionally, trifluridine eye drops and other antivirals may be considered, although efficacy remains uncertain [10]. Topical or oral antibiotics are often used together, either to treat bacterial superinfection or as prophylaxis. The use of topical corticosteroids to control ocular inflammation could unintentionally worsen disease progression by contributing to virus persistence when the corneal epithelium is compromised [10].

## Conclusion

In conclusion, ocular disease has emerged as an increasingly common yet underreported manifestation of mpox in Nigeria since the 2022 global outbreak. Ongoing surveillance and research are vital to better understand eye-related complications and to improve the prevention and treatment of ocular manifestations, thereby preventing significant vision loss. Ophthalmologists should be part of the multidisciplinary team of specialists caring for mpox patients with ocular manifestations.
